# Modifications and Applications of Metal-Organic-Framework-Based Materials for Photocatalysis

**DOI:** 10.3390/molecules29245834

**Published:** 2024-12-11

**Authors:** Weimin Ma, Liang Yu, Pei Kang, Zhiyun Chu, Yingxuan Li

**Affiliations:** MIIT Key Laboratory of Critical Materials Technology for New Energy Conversion and Storage, School of Chemistry and Chemical Engineering, Harbin Institute of Technology, Harbin 150001, China; 44845@sust.edu.cn (W.M.); 24s025026@stu.hit.edu.cn (L.Y.); 2021111145@stu.hit.edu.cn (P.K.); 2021110948@stu.hit.edu.cn (Z.C.)

**Keywords:** metal–organic framework, modifications, applications, photocatalysis

## Abstract

Metal–organic frameworks (MOFs) represent a category of crystalline materials formed by the combination of metal ions or clusters with organic linkers, which have emerged as a prominent research focus in the field of photocatalysis. Owing to their distinctive characteristics, including structural diversity and configurations, significant porosity, and an extensive specific surface area, they provide a flexible foundation for various potential applications in photocatalysis. In recent years, researchers have tackled many issues in the MOF-based photocatalytic yield. However, limited light adsorption regions, lack of active sites and active species, and insufficient efficiency of photogenerated charge carrier separation substantially hinder the photocatalytic performance. In this review, we summarized the strategies to improve the photocatalytic performance and recent developments achieved in MOF and MOF-based photocatalysis, including water splitting, CO_2_ conversion, photocatalytic degradation of pollutants, and photocatalytic nitrogen fixation into ammonia. In conclusion, the existing challenges and prospective advancements in MOF-based photocatalysis are also discussed.

## 1. Introduction

With the advancement of industrialization and the large consumption of fossil energy, such as petroleum, coal, and natural gas, environmental and energy problems have become increasingly prominent. Human beings are faced with severe challenges posed by nature [1,2,3,4,5,6,7]. The pursuit of sustainable energy is a viable strategy to tackle the current energy crisis and environmental challenges by transforming inexhaustible solar energy into renewable clean fuel [6,7,8], which is a sustainable and clean source of energy. The conversion of sunlight into electric or chemical energy is an imperative matter for humanity. Since the discovery by Fujishima and Honda in 1972 of utilizing TiO_2_ photocatalyst for solar-driven water splitting to generate hydrogen [9], a great deal of effort has been invested into the search for efficient and stable photocatalysts, resulting in remarkable advancements. Up to now, a series of semiconductor materials have been developed, such as TiO_2_ [10,11], CdS [12,13,14], g-C_3_N_4_ [15,16,17,18], and Bi_2_S_3_ [19,20,21]. However, the photocatalytic activity of a traditional semiconductor is usually limited by the light absorption range and the efficiency of photogenerated carrier separation, and it is not ideal for the development to meet the energy demand and put it into industrial production; thus, developing new and efficient photocatalytic materials to replace traditional photocatalytic materials is imperative. Therefore, it is necessary to develop photocatalysts with a broad spectral light response and improve the separation efficiency of photogenerated carriers through microstructure regulation to achieve efficient photocatalytic reactions.

Metal–organic matrix materials (MOFs) are composed of metal cations or metal clusters coordinated with various organic connectors, resulting in a porous network structure in one, two, or three dimensions that exhibit a high specific surface area and porosity [22,23,24,25,26]. These materials have demonstrated promising applications and extensive utilization in gas separation [27], sensing [28,29], catalysis [30,31,32,33,34,35,36], drug delivery [37,38,39,40], and other fields. In comparison with traditional semiconductor materials, MOFs have been considered as a novel type of photocatalyst owing to the following advantages: (1) intrinsic pores and high specific surface area shortened the transport distance of charge carrier, and facilitate the diffusion efficiency of reactants and products, and significantly inhibit the recombination of photoexcited electron–hole pairs; (2) tunable structural components, can flexibly design and adjust the ligand to extend the light absorption region and investigate the interactions between different metals and MOF supports; (3) most importantly, MOFs possess a highly porous structure, allowing for the introduction of photosensitizers or cocatalysts [41,42], such as polyoxometalates (POMs) [43], metal nanoparticles [35,44,45,46], and semiconductors [47,48,49], which enhance the effective utilization of photogenerated electrons and holes, minimizing their recombination and thereby enhancing photocatalytic performance.

**Figure 1:**
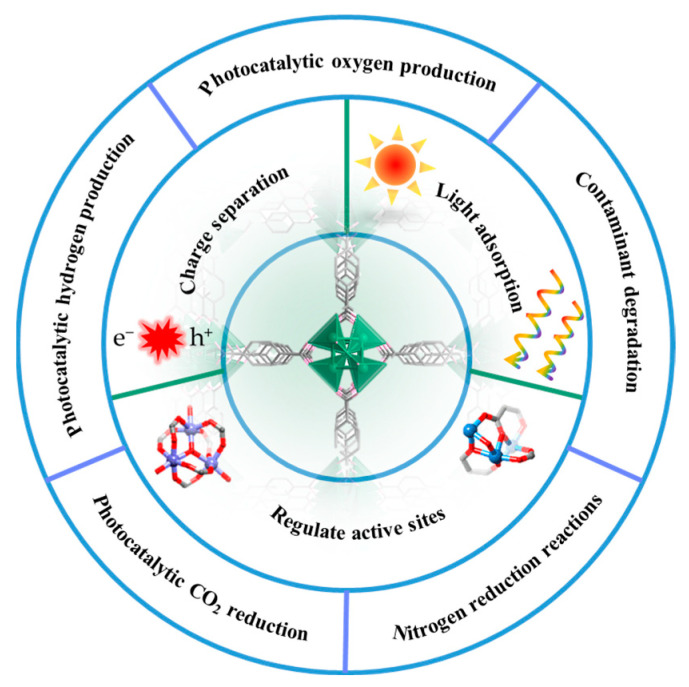
Schematic diagram of MOFs structure, regulation strategies, and their catalytic applications.

In order to enhance the catalytic performance of individual components, researchers have shifted their focus towards synthesizing composite materials, which can compensate for the limitations of their constituent components while leveraging their advantages, thereby exhibiting a broader range of potential applications. Herein, this review systematically provides recent development of MOF-based materials for photocatalysis, including NPs/MOFs [50,51,52,53,54], semiconductor/MOFs [55,56,57,58], and MOF-derived oxide or carbon composites [59,60,61,62]. Furthermore, we elaborately summarized the latest and most significant progress made in the field of photocatalytic water splitting, CO_2_ reduction, contaminant degradation, and nitrogen fixation into ammonia. Specifically, we analyzed the advancements in catalyst design, reaction mechanisms, efficiency improvement, and the potential applications of these technologies in addressing global energy and environmental challenges (Figure 1). Particularly, the current challenges and further development toward MOF in the photocatalytic field are also highlighted.

## 2. Strategies to Improve Photocatalytic Performance

### 2.1. Light Adsorption

Light absorption is the primary requirement in photocatalytic reactions. Sunlight consists of 4% ultraviolet light, 45% visible light, and 50% infrared light, with a significantly higher proportion of visible and infrared light compared to UV light [63]. Therefore, it is imperative to expand the adsorption range to visible and near-infrared regions, which enables more efficient utilization of sunlight. Consequently, extensive investigations have focused on broadening the light absorption capabilities of MOF materials for effective solar energy utilization.

Organic linker modulation can efficiently broaden the range of light harvesting. Silva and co-workers introduced the amino group into UiO-66 to prepared UiO-66-NH_2_, by replacing H_2_BDC with NH_2_-H_2_BDC [64]. The absorption band edge of UiO-66-NH_2_ is extended from 300 nm to a wide absorption range from 300 to 440 nm with the incorporation of an amino group. Compared with UiO-66-NH_2_, UiO-66 could not detect hydrogen generation, while UiO-66-NH_2_ could detect hydrogen generation. Although the quantum yield of 3.5% is not ideal, this work sets a precedent for MOF modification of organic ligands for photocatalytic hydrogen production, and also provides technical support for the subsequent use of MOFs for visible light catalytic hydrogen production.

**Figure 2:**
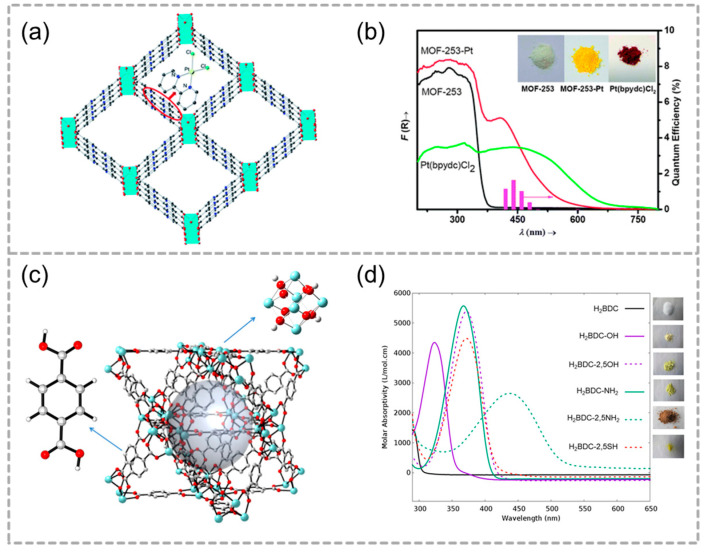
(**a**) Model structure of MOF-253-Pt. (**b**) UV–vis spectra of MOF-253, MOF-253-Pt, and Pt(bpydc)Cl_2_ and the corresponding quantum efficiencies of hydrogen evolution for MOF-253-Pt at different wavelengths. The inset shows the colors of the samples. Adapted with permission from RSC [50]. (**c**) Structure of the UiO-66 framework. (**d**) Experimental linker spectra and photographs of the pure linkers. Reproduced with permission from ACS [65].

Apart from organic ligands, metal ions, especially noble metal nanostructures, garnered significant attention because of their distinctive characteristics, including large optical field enhancement functions, leading to strong light scattering and absorption. For this purpose, Zhou and his partners chose a robust framework (MOF-253) as a prototype, and then implanted a platinum complex in MOF-253 to obtain a similar MOF-253-Pt complex (Figure 2a) [65]. The color of MOF-253 turned from white to bright yellow with Pt loaded. Obviously, the absorption range of MOF-253-Pt is wider than MOF-253, that is to say, MOF-253-Pt exhibits a unique absorption band at 410 nm and an extended edge to 650 nm due to PtCl_2_ binding with bipyridine (Figure 2b). The functionalized MOF-253-Pt is an effective photosensitizer and photocatalyst for hydrogen evolution, exhibiting a five-times-greater activity than its corresponding complex under visible light.

Rare-earth elements play an important role in various fields and are attracting more and more attention from scientists. Xu and co-workers reported that the modification of MOFs with rare-earth elements can broaden the absorption spectrum [33], and the electrons present in the unoccupied 4f orbital can serve as traps for photo-excited carriers, preventing the recombination of electron–hole pairs and thereby enhancing photocatalytic activity.

In order to investigate the effect of organic ligands on the light absorption of photocatalysts, UiO-66-X was used as a model, a combination of theoretical and experimental studies was conducted. Kevin Hendrickx and co-workers investigated the single- and double-functionalized linker (X = OH, NH_2_ (or SH)) to gain a more comprehensive understanding of the functionalization choices (Figure 2c). Calculations using static time-dependent density functional theory for the linker were integrated with molecular dynamics simulations, and these findings were contrasted with experimental UV–vis absorption spectra to clarify the electronic effects on the absorption properties. In comparison to the single-substituted variant, the double-substituted linker showed a more significant shift (Figure 2d), indicating its potential as a candidate for further investigation in photocatalysis. Subsequently, the interaction between the organic linker and the inorganic component of the framework was theoretically analyzed using cluster models. The study identified the ligand-to-metal charge transfer influenced by functionalization, revealing that linker modifications can adjust the band gap of UiO-66 from 4.0 to 2.2 eV. The periodic density of the examined state may elucidate the modulation of the band gap within the framework, which was influenced by functionalization occurring in the original UiO-66 host’s band gap.

### 2.2. Regulate Active Sites

**Figure 3:**
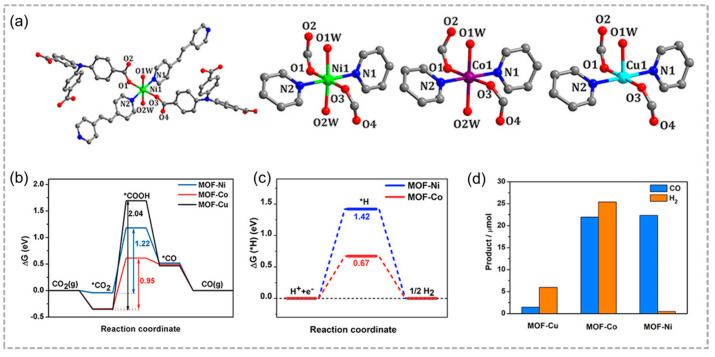
(**a**) Coordination environment and (**b**) free energy diagram of CO_2_RR for MOF–Ni, MOF–Co, and MOF–Cu catalysts. (**c**) Free energy diagram of HER. (**d**) Yield of CO and H_2_ produced by photocatalytic reduction of CO_2_ by MOF–Cu, MOF–Co, and MOF–Ni. * = the active species. Adapted with permission from ACS [66].

Ligands and secondary building units are essential in MOFs, with metal ions or clusters serving as the primary active sites for photocatalysis. Recently, Lan and co-workers synthesized three stable isostructural MOFs by manipulating the types of metal ions (Ni, Co, and Cu) (Figure 3a), and then investigated the properties of photocatalytic CO_2_ reduction [66]. CO_2_ adsorption is the most critical step in the subsequent conversion, in which Co and Cu have a weak adsorption capacity for CO_2_, while MOF-Ni has strong binding with CO_2_, so MOF-Ni shows the highest activity and CO selectivity (Figure 3b). Furthermore, MOF-Ni has a high free energy barrier for H_2_ evolution (Figure 3c). The results revealed that the Ni(II) ion acted as an effective active site for CO_2_ reduction (Figure 3d). The mixed-metal strategy was achieved by adding new active centers into MOFs, which is an effective strategy to enhance the efficiency of photogenerated electron and hole separation, thereby boosting photocatalytic activity.

Ye and co-workers achieved modular optimization in MOFs by integrating coordinatively unsaturated single atoms into the porphyrin center, resulting in the metallization of Zn or Co to form new structures, MOF-525-Zn and MOF-525-Co [67]. It is worth noting that the newly MOF-525-Co shows the ability of selective adsorption and efficient conversion of CO_2_ to CO under visible light irradiation, which is much higher than that of modified MOF-525-Zn and the parent MOF-525. The difference in activity between MOF-525-Zn and MOF-525-Co is due to the separation efficiency of photogenerated electrons and holes, and further analysis is due to the different metals located in the porphyrin units. MOF-525-Co and MOF-525-Zn, which possess distinct active sites, enhance separation efficiency by promoting energy transfer from the porphyrin units to the lower-energy Co or Zn trap sites, thereby optimizing the electron transfer pathways to Zr oxo-clusters. Importantly, the current intensity observed in MOF-525-Co significantly exceeds that of MOF-525-Zn, and the diversity in catalytic active sites, and thus, accounts for different catalytic activity.

### 2.3. Charge Carrier Separation

The separation of charge carriers is also a crucial criterion for photocatalysis. The photocatalyst generates photo-induced electrons and holes upon photon absorption, driving the redox reactions and converting solar energy into chemical energy. In recent years, the emergence of MOFs has sparked a surge in research on utilizing these materials as photocatalysts. However, current studies indicate that the photocatalytic efficiency of MOF materials consistently falls short compared to traditional photocatalysts. The main issue is the ineffective separation of photogenerated electron–hole pairs in MOF materials, resulting in significantly shortened lifetimes for these species and subsequently diminishing the photocatalytic performance.

The piezoelectric effect generates an internal electric field that has the potential to enhance transport routes of photogenerated charges during photocatalytic processes, thereby enhancing the photocatalytic activity. Fang and co-workers developed a novel piezoelectric photocatalytic heterojunction UiO-66-NH_2_@CdS [68], which was approximately four-times higher compared to the absence of ultrasound. The photocatalytic mechanism shows that piezoelectric polarization distorts the energy band of MOF, modifies charge transfer pathways, and mitigates electron–hole pair recombination, thereby enhancing hydrogen evolution performance. Recently, Jiang and co-workers synthesized UiO-66-NH_2_(Zr) and UiO-66-NH_2_(Hf), two isostructural MOFs with significantly different piezoelectric characteristics. Consequently, UiO-66-NH_2_(Hf) demonstrates approximately a 2.2-times-greater activity than UiO-66-NH_2_(Zr) under identical conditions [34].

**Figure 4:**
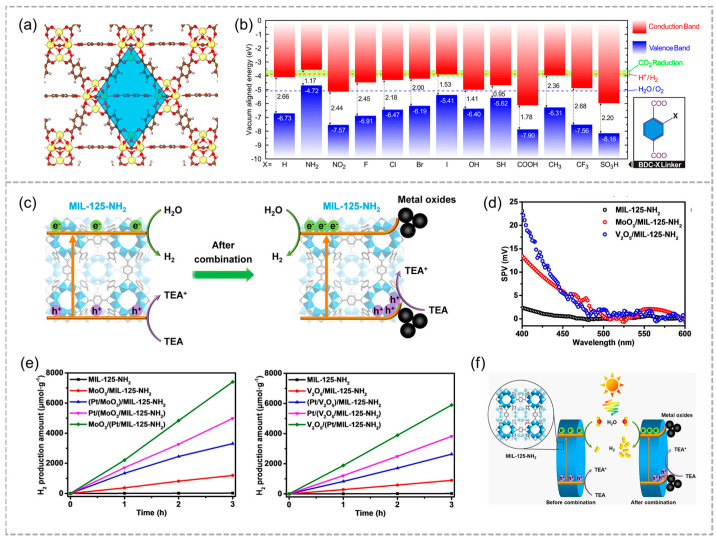
(**a**) Framework structure of UiO–66. (**b**) The band alignment of UiO–66(Ce)–X, with the structure of the BDC–X linkers displayed in the in the box at lower right. Adapted with permission from ACS [69]. (**c**) Schematic diagram of photocatalytic H_2_ generation before and after contact with MIL–125–NH_2_ and metal oxides. (**d**) SPV spectra curves of MIL–125–NH_2_, MoO_3_/MIL–125–NH_2_, and V_2_O_5_/MIL–125–NH_2_. (**e**) Photocatalytic H_2_ generation rates for Pt–loaded MoO_3_/MIL–125–NH_2_ and V_2_O_5_/MIL–125–NH_2_ catalysts at different positions. (**f**) Schematic diagram of photocatalytic H_2_ production before and after combination. Adapted with permission from WILEY [58].

Band bending is essential for the separation of photogenerated carriers. Wu and his partners proposed that the incorporation of cerium (Ce) into MOF materials can promote the separation efficiency of electron–hole pairs (Figure 4a) [69]. The classical MOF material UiO-66 (whose metal elements can be Zr, Hf, Th, Ti, U, or Ce) is calculated by density functional theory, and the ligand-to-metal charge transfer, which can separate the hole pairs of photogenerated electrons is found. The LMCT process is only thermodynamically advantageous in UiO-66(Ce) materials because Ce^4+^ in this material has a low-energy 4f empty orbital, which can be used to receive photogenerated electrons. Modifying the ligand (linker) of the UiO-66(Ce) material with groups such as NH_2_ can further regulate the electronic structure of the material (Figure 4b), improving its efficiency for certain specific photocatalytic reactions, such as photolytic water to produce hydrogen.

Recently, Jiang and co-workers first combined the metal oxides (MoO_3_ and V_2_O_5_) with MIL-125-NH_2_ (Figure 4c), leading to the improvement in the efficiency of charge carriers by establishment of a built-in electric field [58]. The MOF composites present higher photocatalytic H_2_ production activities than that of the pristine MOF. In comparison to MIL-125-NH_2_, the MoO_3_/MIL-125-NH_2_ and V_2_O_5_/MIL-125-NH_2_ composites exhibit notably stronger SPV signals, indicating a higher concentration of hole accumulation on the MOF surface (Figure 4d), while electrons migrate to Pt sites to facilitate proton reduction (Figure 4e). This finding clearly indicates that band bending takes place in the MOF, when it is combined with the oxides. In short, the deposition of Pt on the MOF further enhances the catalytic activity by facilitating electron transfer via the bent band (Figure 4f).

## 3. Applications for Photocatalysis

### 3.1. Photocatalytic Hydrogen Evolution (HER)

H_2_ is a green renewable energy, and is the most ideal fuel to meet future energy demands. Solar-powered water decomposition to produce hydrogen is a promising approach to address the growing energy crisis and environmental problems. Photocatalytic cracking of water for hydrogen production has the following advantages: (1) Water resources as the most abundant and cleanest source on earth, serves as an ideal raw material. (2) The combustion of hydrogen yields a substantial amount of energy with environmentally friendly implications, and possesses a three-times-higher calorific value than that of gasoline [70]. (3) Utilizing clean and sustainable solar energy directly converts sunlight into hydrogen, thus establishing an efficient energy recycling platform, which is attractive for achieving human energy goals.

**Figure 5:**
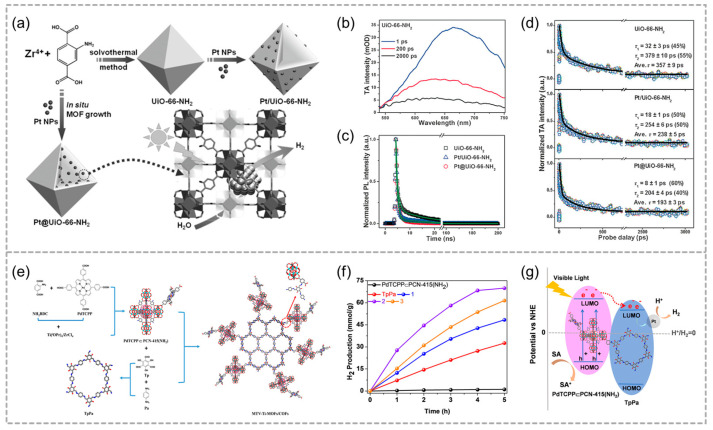
(**a**) A schematic representation depicts the synthesis of Pt@UiO-66-NH_2_ and Pt/UiO-66-NH_2_, highlighting the photocatalytic hydrogen production process occurring over Pt@UiO-66-NH_2_. (**b**) TA spectra of UiO-66-NH_2_ (excitation at 400 nm) with TA signal given in mOD (OD: optical density). (**c**) Time-resolved PL decay profiles and (**d**) TA kinetics for UiO-66-NH_2_, Pt@UiO-66-NH_2_, and Pt/UiO-66-NH_2_, respectively. Reproduced with permission from WILEY [51]. (**e**) The schematic synthesis of MTV-Ti-MOF/COF. (**f**) The photocatalytic H_2_ evolution activities. (**g**) The photocatalytic H_2_ evolution mechanism. Reproduced with permission from WILEY [52].

As a co-catalyst, Pt NPs are essential for charge separation and transport. Do the spatial distribution of Pt NPs and the relative position of MOFs have different catalytic effects? Due to its excellent porosity and adjustable pore structure, MOF materials have been widely used in photocatalytic water decomposition. The confined Pt NPs not only reduce the overpotential for hydrogen generation but also improve charge separation by creating a heterogeneous junction with the MOF support. Jiang and co-workers confined Pt NPs inside or loaded on the surface of the UiO-66-NH_2_, named as Pt@UiO-66-NH_2_ and Pt/UiO-66-NH_2_, respectively (Figure 5a) [51]. After introduction of Pt NPs, UiO-66-NH_2_ catalysts show superior photocatalytic performance for H_2_ production by enhancing electron transfer and suppressing charge recombination (Figure 5b–d). In that case, the photocatalytic efficiency correlates mainly with the position of decorated Pt. The Pt@UiO-66-NH_2_ markedly shorten the electron transfer distance from the MOF to Pt NPs, thereby improving charge-carrier utilization and leading to significantly enhanced hydrogen production in comparison to Pt/UiO-66-NH_2_.

Titania (TiO_2_), as the most classical photocatalyst, has been widely used in the field of photocatalytic hydrogen production via its high effectiveness and abundance. Ma and coworkers synthesized and utilized a series of multivariate Ti-MOF/COF hybrid materials for visible-light-driven photocatalytic H_2_ production (Figure 5e) [52]. Composite 2 achieves a H_2_ evolution rate of 13.98 μmol g^−1^ h^−1^, significantly outperforming both PdTCPP⸦PCN-415-(NH_2_) and TpPa (Figure 5f), highlighting the benefits of covalent linking. The covalent linkage between PdTCPP⸦PCN-415-(NH_2_) and TpPa enables efficient separation of photogenerated electrons and holes (Figure 5g), facilitating H^+^ reduction by Pt nanoparticles and hole capture by the electron acceptor SA, thereby completing the catalytic cycle.

**Figure 6:**
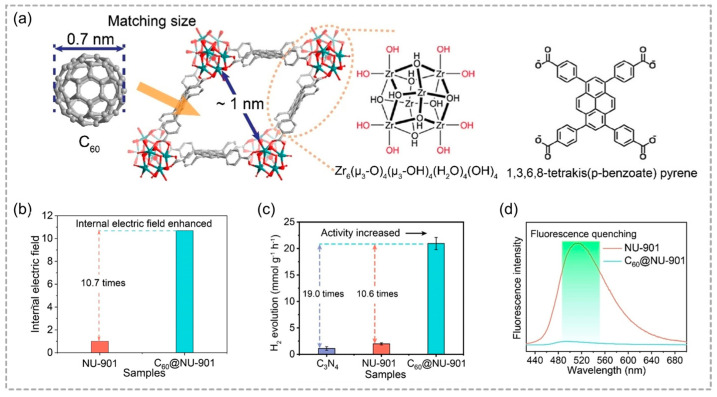
(**a**) Immobilization of C_60_ within the pores of NU–901. (**b**) The internal electric field intensity for NU–901 and C_60_@NU–901. (**c**) Photocatalytic hydrogen evolution rates for C_3_N_4_, NU–901, and C_60_@NU–901. (**d**) The photoluminescence spectra. Reproduced with permission from WILEY [53].

Zhu and co-workers relied on the host–guest interactions and uneven charge distribution strategy, and constructed a novel MOF photocatalyst (C_60_@NU-901) by encapsulating C_60_ into a size-matched zirconium-based MOF, NU-901 [53]. By virtue of a robust built-in electric field, C_60_@NU-901 tends to demonstrate a 10.7- and 469-times-higher photocatalytic hydrogen evolution activity than that found in benchmark NU-901 and C_60_ powder, achieving a maximum rate of 22.3 μmol g^−1^ h^−1^ under visible light irradiation (Figure 6). Correspondingly, the incorporation of C_60_ enhances exciton dissociation and free charge carrier generation, while the inherent electric field promotes their separation and transfer. Consequently, C_60_@NU-901 combined with high electron–hole separation efficiency and multi-path charge transfer, thereby significantly elevating the overall photocatalytic efficacy. The notion of enhancing charge separation via host–guest interactions offers a compelling strategy for the advancement of photocatalyst design, and realizes the efficient transformation of renewable solar energy into sustainable energy.

### 3.2. Photocatalytic Oxygen Reaction (OER)

In recent years, photocatalytic water decomposition is gaining attention for its direct conversion of solar energy into green renewable fuel. However, photocatalytic water oxidation is the rate-determining step in the process of water splitting to generate oxygen. The water oxidation reaction has slower kinetics compared to the hydrogen evolution half reaction both in natural and artificial photosynthesis due to factors such as high over-potential, multiple-electron transport, and the formidable energy barrier associated with O-O formation. Consequently, progress in developing efficient catalysts for the water oxidation half-reaction lags behind that of photocatalytic hydrogen evolution. Henceforth, improving the performance of photocatalytic water oxidation through simple and effective strategies is a key challenge in the field.

**Figure 7:**
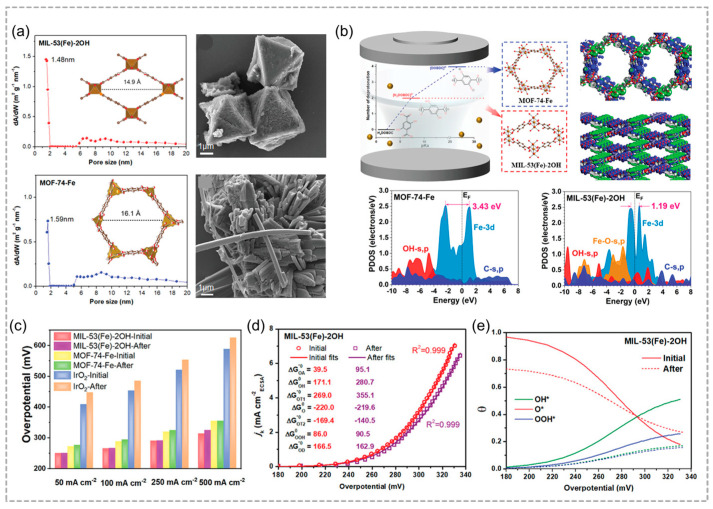
(**a**) Pore size distribution and morphology of MIL–53(Fe)–2OH and MOF–74–Fe catalysts. (**b**) Schematic synthesis of MIL–53(Fe)–2OH and MOF–74–Fe catalysts and the analysis Fe–3d, OH–s,p and C–s,p. (**c**) Overpotential histogram of MIL–53(Fe)–2OH, MOF–74–Fe, and IrO_2_ catalysts before and after reaction at different current densities. (**d**) Electrokinetic current density under optimal kinetic fitting of OER. (**e**) Coverage of O*, OH*, and OOH* intermediates of MIL–53(Fe)–2OH catalyst before and after stability testing at different potentials. Reproduced with permission from WILEY [71].

Yang and co-workers rationally designed and fabricated MIL-53(Fe)-2OH and MOF-74-Fe with and without uncoordinated phenolic hydroxyl groups (Figure 7a) [71], MIL-53(Fe)-2OH with desirable electronic structure and uncoordinated phenolic hydroxyls, demonstrates rapid OER kinetics with a low overpotential of 215 mV and a turnover frequency significantly higher than that of the commercial IrO₂ catalyst (Figure 7b,c). The reduced e_g_-t_2g_ splitting of Fe-3d orbitals and electroactive O sites in MIL-53(Fe)-2OH enhance OER efficiency by decreasing overpotential (Figure 7d). The unique electronic structures of MIL-53(Fe)-2OH enhance the adsorption and desorption of oxygen intermediates, thus improving its catalytic activity and stability for OER compared to MOF-74-Fe (Figure 7e). Su and coworkers investigated three Fe-based MOFs-MIL-101(Fe), MIL-88B(Fe), and MIL-53(Fe)-as photocatalysts for the OER. Among these, MIL-101(Fe) exhibited remarkable photocatalytic efficacy for OER when Na_2_S_2_O_8_ served as the electron acceptor while [Ru(bpy)_3_]^2+^ acted as the photosensitizer. Ultimately, the holes generated by the catalyst were harnessed by water, leading to its oxidation and the generation of O_2_.

### 3.3. Photocatalytic CO_2_ Reduction

#### 3.3.1. Pristine MOF

CO_2_ is the main pollutant that causes the greenhouse effect, which affects climate change. At present, the concentration of CO_2_ in the atmosphere has risen sharply to about 400 ppm, which has brought serious environmental problems. Consequently, mitigating CO_2_ levels in the atmosphere has become urgent. It is necessary to use inexhaustible solar energy as an energy input, so it is imperative to develop photocatalysts that can capture and achieve a CO_2_ reduction reaction (CO_2_RR). MOF composites offer distinct advantages including enhanced CO_2_ adsorption capacity and facile adjustment of reaction selectivity through post-synthesis modification. Thus, the application of MOF composites presents a promising avenue for tackling this challenge.

The transformation of CO_2_ into high value-added fuels like methane (CH_4_), carbon monoxide (CO), and formate (HCOO^−^) embodies a remarkably promising technology that is expected to yield significant advantages in both the energy and environmental domains [59,60,61,63,66,67]. The process of photocatalytic CO_2_ reduction involves the utilization of abundant solar energy to cleave the robust C=O bonds and generate valued chemicals, which offers an ideal solution to the global energy crisis and environmental challenges.

**Figure 8:**
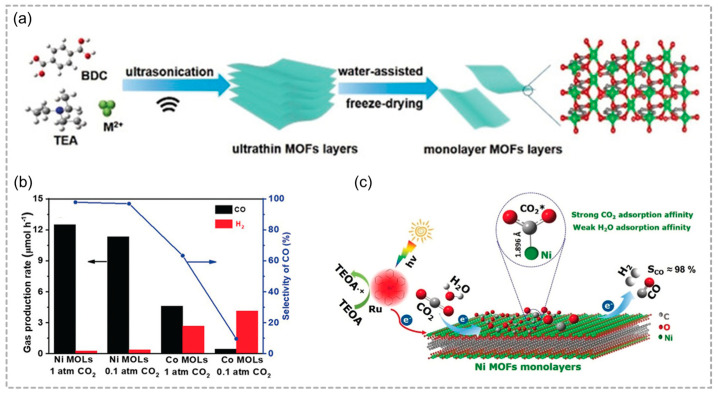
(**a**) The schematic illustration for the preparation of Ni MOLs. (**b**) CO_2_ photoreduction performance over Ni MOLs and Co MOLs in pure CO_2_ and diluted CO_2_ (10%). (**c**) The proposed mechanism delineates the conversion of CO_2_ to CO via Ni MOLs using [Ru(bpy)_3_]^2+^ and TEOA under visible light. Reproduced with permission from WILEY [72].

Combined with a structure–property relationship, the metal sites in MOFs are pivotal for adjusting the photocatalytic CO_2_ reduction performance. Lin and co-workers constructed Ni MOF monolayers (Ni MOLs) for the photoreduction of diluted CO_2_ (Figure 8a) [72]. In a pure CO_2_ environment, Ni MOLs endowed with a wealth of coordinatively unsaturated Ni sites exhibited an impressive apparent quantum yield of 2.2% at 420 nm, coupled with [Ru(bpy)_3_]Cl_2_·6H_2_O as the photosensitizer and triethanolamine as the electron donor achieved an exceptional CO selectivity of 97.8%. Moreover, Ni MOLs achieved an impressive apparent quantum yield of 1.96% and remarkable CO selectivity of 96.8% even in diluted CO_2_ (10%) (Figure 8b). Ni MOLs enhance CO_2_ photoreduction efficiency by favoring strong CO_2_ adsorption and minimizing H_2_O interaction, leading to improved activity and selectivity (Figure 8c).

**Figure 9:**
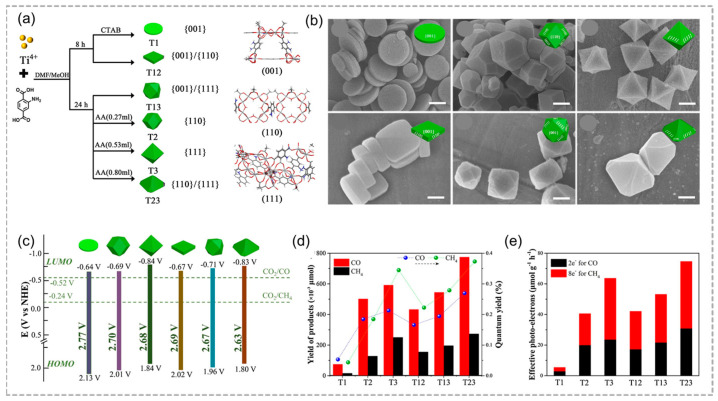
(**a**) Schematic illustration of the morphology and facet over MIL–125–NH_2_(Ti). (**b**) SEM images of as–synthesized MIL–125–NH_2_(Ti) with different shape. Scale bar: 500 nm. (**c**) HOMO–LUMO gap of the as–synthesized MIL–125–NH_2_(Ti). (**d**) The yield of CO and CH_4_ products and (**e**) effective photo–electrons after irradiation for 5 h over the as–synthesized MIL–125–NH_2_(Ti). Adopted with permission from Elsevier [73].

Recently, Sun and co-workers analyzed the influence of six distinct variants of MIL-125-NH_2_(Ti) through the exposure of individual low-index facets as well as the co-exposure of mixed facets on the photoreduction process of CO_2_ (Figure 9a) [73]. The low-index facets {001}, {110}, and {111} display unique morphologies: T1 as a disk-shaped plate, T2 as a rhombic dodecahedron, and T3 as an octahedron, respectively (Figure 9b). The heterojunction at the MIL-125-NH_2_(Ti) facet not only enhances light absorption but also accelerates the charge migration (Figure 9c). Due to the surface heterojunction formation, MIL-125-NH_2_(Ti) with co-exposed {110}/{111} facets achieves optimal CO and CH_4_ production rates, 10- and 18-times higher than T1 with only the {001} facet, and thus facilitates the interfacial charge transfer (Figure 9d,e). Therefore, regulating and optimizing facets are essential for photocatalytic performance, aiding in the initiation of reactions, promoting electron migration, and inhibiting charge recombination to enhance CO_2_ reduction.

#### 3.3.2. MOF Composite

Coupling MOFs with conventional catalysts such as metal nanoparticles, semiconductor materials, or light-harvesting molecules to prepare composite materials is an effective way to improve the activity of photocatalytic conversion of CO_2_. MOFs with tunable porosity not only can enhance light absorption by modifying the arrangement and density of their active sites, but also allow for the optimization of pore size and surface area, and thus facilitate the diffusion and accessibility of reactant molecules to the active catalytic sites. By leveraging these advantages, MOFs with tunable porosity not only enhance photocatalytic activity but also expand the scope of applications, making them a versatile class of materials in sustainable catalysis. Combining conventional catalysts and MOFs in CO_2_ photocatalytic reduction effectively regulates active sites, accelerates carrier separation and migration, and enhances visible light absorption. In addition to light and CO_2_ absorption capacity, the efficiency of spatial separation of photo-generated electrons and holes is a crucial index. In general, encapsulated metal nanoparticles (MNPs) are used as electron acceptors, which can not only promote the transfer of photogenerated electrons from MOFs to MNPs, but also promote the electron–electron pore transfer by constructing type II heterojunction or type z heterojunction, thereby improving the photocatalytic performance.

**Figure 10:**
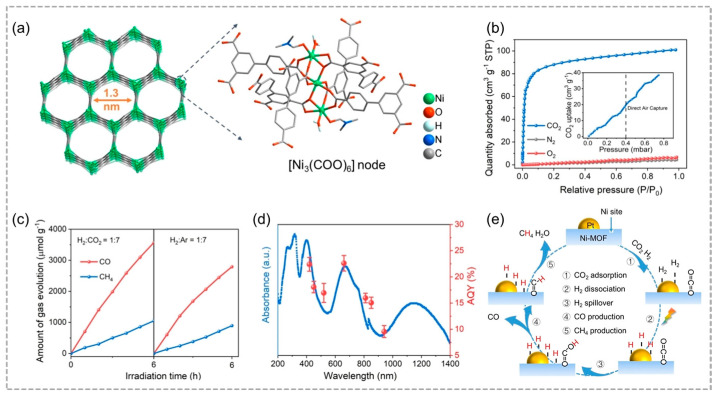
(**a**) Three-dimensional frame structure diagram of Ni–MOF and the corresponding trinuclear [Ni_3_(COO)_6_] node. (**b**) CO_2_, N_2_, and O_2_ adsorption curves of Pt/Ni–MOF at 25 °C, insert: CO_2_ absorption at a pressure of 0.4 mbar (the pressure pertinent to direct air capture). (**c**) Thermal-photocatalytic yields of CO and CH_4_ on the pristine Pt/Ni–MOF in H_2_: CO_2_ (with a 1:7 ratio) and on Pt/Ni–MOF with captured ambient CO_2_ in H_2_: Ar (with a 1:7 ratio), respectively. (**d**) AQYs of Pt/Ni–MOF for CO_2_ reduction under different wavelengths. (**e**) Schematic diagram of the proposed pathway for Pt/Ni–MOF photocatalytic conversion of CO_2_. Reproduced with permission from WILEY [54].

Directly capturing and converting ultralow concentrations of atmospheric CO_2_ via photocatalytic route are highly required, and this sustainable technology holds great potential to achieve carbon neutrality in the future. The in situ transformation of captured CO_2_ eliminates energy costs, infrastructure needs, and expenses associated with CO_2_ separation while ensuring effective conversion of even trace amounts of CO_2_ from the air into valuable chemicals. Based on this concept, Li and co-workers created a novel Ni-MOF integrated with Pt (Pt/Ni-MOF) that selectively captures and concentrates atmospheric CO_2_ (Figure 10a), demonstrating remarkable efficiency in the thermal-photocatalytic conversion of CO_2_ with H_2_, even when exposed to infrared light [54]. The synergistic interaction between the dual-active sites of Pt and Ni enhances catalytic performance by facilitating non-competitive adsorption of H_2_ and CO_2_, with Ni promoting CO_2_ activation and photogenerated electrons driving H_2_ dissociation at the Pt sites (Figure 10b,c). Concurrently, thermal energy facilitates the migration of dissociated H_2_ to Ni sites, where adsorbed CO_2_ is thermally reduced to produce CO and CH_4_. Due to the metallic property of Pt/Ni-MOF, the catalyst achieved an unprecedented 9.57% efficiency at 940 nm for atmospheric CO_2_ conversion (Figure 10d), enabling independent CO_2_ procurement and improving energy efficiency by eliminating the need for regeneration of capture media and release of molecular CO_2_ (Figure 10e).

**Figure 11:**
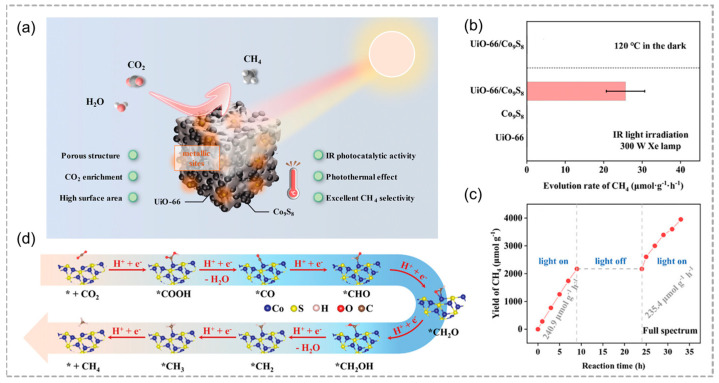
(**a**) A schematic illustration demonstrates the effective reduction of CO_2_ to CH_4_ using an IR–light–driven UiO–66/Co_9_S_8_ composite catalyst, leveraging the advantages of both porous UiO–66 and metallic Co_9_S_8_. (**b**) CH_4_ evolution rates of CO_2_ photoreduction over different catalysts. (**c**) Stability assessments of UiO–66/Co_9_S_8_ for photocatalytic activity under full–spectrum light irradiation. (**d**) A possible reaction pathway for photocatalytic CO_2_ methanation, * = active site. Reproduced with permission from WILEY [55].

Infrared light, accounting for approximately 50% of the solar spectrum, is not effectively utilized. To solve this problem, metallic photocatalysts without energy gaps to enhance infrared light absorption for CO_2_ conversion were investigated. Another great challenge is to achieve single-product selectivity for photocatalytic CO_2_ reduction. Xu and co-workers coupled the metallic nature of Co_9_S_8_ and the local CO_2_ concentrated capacity of UiO-66 to design a metallic photocatalyst UiO-66/Co_9_S_8_ composite (Figure 11a) [55], which shows observable IR-light photocatalytic CO_2_-to-CH_4_ conversion. UiO-66/Co_9_S_8_ produces CH_4_ at a rate of 25.7 μmol g^−1^ h^−1^ with nearly 100% selectivity under infrared light (Figure 11b,c). It is worth mentioning that Co_9_S_8_ features two distinct Co sites: Co1 with high electron density and Co2 with low electron density. The formation of *COOH is the rate-limiting step at both sites; however, Co1 has a lower energy for *COOH formation, facilitating further protonation of *CO and resulting in exceptionally high selectivity for CH_4_ (Figure 11d).

#### 3.3.3. MOF Derived Materials

Photothermal hydrogenation of CO_2_ represents a promising approach to address energy and environmental challenges, but the photothermal CO_2_ hydrogenation reaction still needs to develop highly efficient and durable catalysts. MOF materials are considered as promising precursors or sacrificial templates for derived functional materials, such as metal NPs, metal oxides, metal sulfides, and their heterostructures, under the corresponding treatments. These derived materials typically retain the morphology and porosity characteristics of MOFs, accelerating the effective mass transport and charge transfer in catalytic processes, thereby leading to enhanced catalytic activity.

The direct transformation of CO_2_ into CH_4_ provides an eco-friendly method to reduce the concentration of CO_2_ in the atmosphere, thereby reducing the dependence on non-renewable energy sources. The process is hindered kinetically at low temperatures due to high stability and reactive inertia of CO_2_. However, on a thermodynamic level, CO_2_ methanation is an exothermic reaction, and thus, it is imperative to lower the reaction temperature. Hu and co-workers developed the core–shell Co/MnO@PGC catalyst from calcinated bi-metal CoMn-MOF-74, which possesses abundant defects and oxygen vacancies and thus enhances CO_2_ adsorption and activation, enabling CH_4_ formation with over 99% selectivity and a STY_CH4_ of 0.14 μmol CH_4_ s^−1^ gcat^−1^ even at 160 °C [74]. The improved performance of Co/MnO@PGC can be ascribed to the synergistic interaction between Co^0^ and MnO at their heterointerface. Specifically, H_2_ is efficiently dissociated on Co^0^, while CO_2_ undergoes strong adsorption and activation on the adjacent MnO.

**Figure 12:**
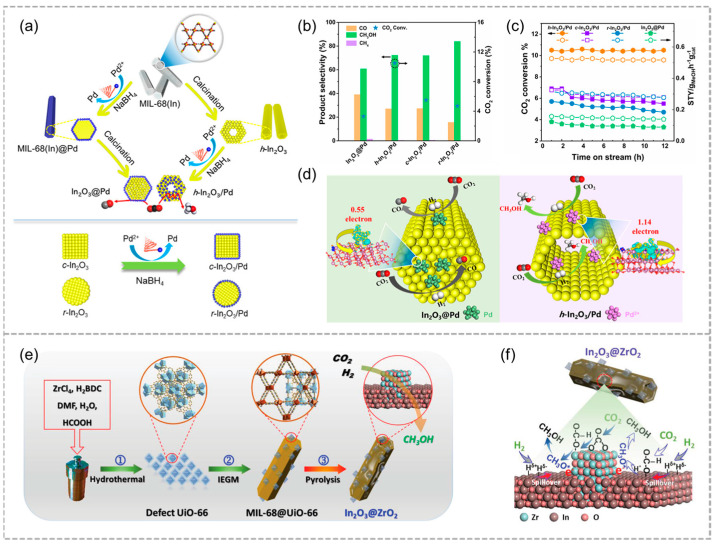
(**a**) Diagrammatic representation of the synthesis process for In_2_O_3_@Pd and h-In_2_O_3_/Pd catalysts (above) as well as c-In_2_O_3_/Pd and r-In_2_O_3_/Pd catalysts (below). (**b**) The conversion of CO_2_ and the distribution of products across four different Pd/In_2_O_3_ catalysts. (**c**) Time on stream (TOS) for the four catalysts in CO_2_ hydrogenation under standard conditions. (**d**) Schematic illustration of CO_2_ hydrogenation to methanol. Reproduced with permission from ACS [59]. (**e**) Schematic illustration of the synthetic process of hollow-structured In_2_O_3_@ZrO_2_ heterostructure. (**f**) Simplified model showing the main reaction mechanism on In_2_O_3_@ZrO_2_ catalyst. Reproduced with permission from WILEY [60].

In addition to converting CO_2_ into gaseous products such as CO and methane, it is more attractive to convert CO_2_ into higher value-added chemicals, such as methanol (CH_3_OH), which is an important intermediate in chemical production. Huang and co-workers employed MIL-68(In) nanorods to synthesize hollow In_2_O_3_ nanotubes (h-In_2_O_3_) and prepare supported Pd catalysts for CO_2_ hydrogenation into methanol (Figure 12a) [59]; this result demonstrated that manipulating In_2_O_3_ morphology significantly affects the SMSI between Pd and In_2_O_3_, inducing different electronic states of Pd. Notably, the performance of Pd loaded on h-In_2_O_3_ was superior to that of In_2_O_3_ with other morphologies. This study demonstrates that regulating In_2_O_3_ morphology not only affects the electronic properties of Pd, but also affects the SMSI with In_2_O_3_, which are key factors contributing to enhanced catalytic performance (Figure 12b–d). Recently, Hu and co-workers developed a hollow In_2_O_3_@ZrO_2_ heterostructure through pyrolysis of MIL-68@UiO-66, achieving enhanced activity and stability for CO_2_ hydrogenation to methanol (Figure 12e). At the In_2_O_3_/ZrO_2_ heterointerfaces, electrons transfer from ZrO_2_ to In_2_O_3_, facilitating H_2_ dissociation and enabling the hydrogenation of formate and methoxy species into methanol through a specific reaction pathway: HCOO* → CH_3_O* → CH_3_OH* → CH_3_OH (g) (Figure 12f) [60].

**Figure 13:**
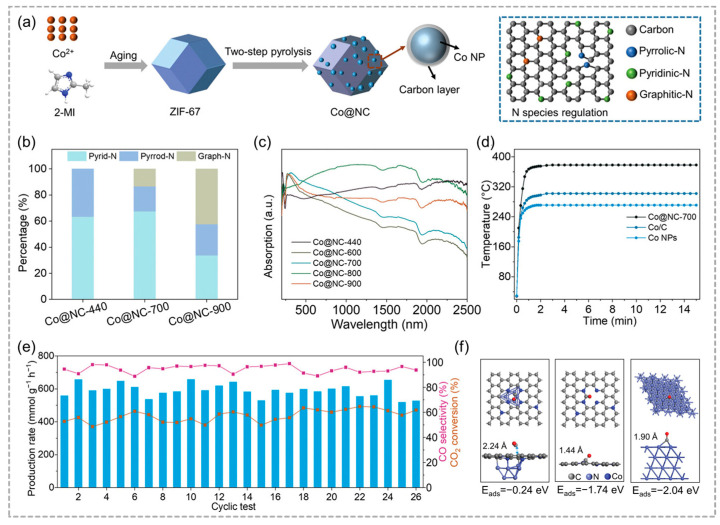
(**a**) Schematic diagram of the preparation of the nitrogen-doped carbon layer-coated cobalt nanoparticles catalyst (Co@NC). (**b**) Nitrogen species and content distribution of catalytic pyrolysis at different temperatures. (**c**) UV–Vis–NIR absorption spectra for the synthesized Co@NC catalysts. (**d**) Monitoring of photothermal temperatures for Co@NC-700, Co/C, and uncoated Co nanoparticles under a light intensity of 3.0 W cm^−2^. (**e**) Photothermal CO_2_ hydrogenation stability evaluation for the Co@NC-700 with Xe irradiation at 3.0 W cm^−2^. (**f**) Optimized configuration for CO adsorption on Co_9_@graphene, graphene, and the Co (111) surface. Reproduced with permission from WILEY [61].

The long-term stability and cyclic stability of catalysts are also important indicators to evaluate the catalysts. Photocatalysts utilizing metal nanoparticles derived from MOFs have exhibited outstanding efficacy in photocatalytic CO_2_ reduction processes. Ye and co-workers utilized MIL-101 as the precursor and developed an Fe@C nanostructure where plasmonic Fe nanoparticles were coated with ultrathin carbon layers [75]. The resulting Fe@C nanostructure was capable of absorbing the entire UV–Vis–NIR spectrum and acted as a catalyst for the solar-driven conversion of CO_2_ into CO. The strong absorption of visible light and infrared radiation generated a notable thermal effect that facilitated the reaction (Figure 13c). Ultraviolet light enhanced the local surface-plasmon resonances of iron can activate nonpolar CO_2_ molecules and facilitate the desorption of generated CO, resulting in improved solar-driven conversion of CO_2_ to CO with the Fe@C photocatalyst. Xiong and co-workers developed a range of N-doped carbon-coated Co nanoparticles (Co@NC) as photothermal catalysts by optimizing nanoparticle size, carbon layer thickness, and the type of nitrogen doping through pyrolysis of the ZIF-67 precursor (Figure 13a) [61]. The Co@NC-700 catalyst shows the exceptional activity and stability for photothermal CO_2_ hydrogenation to CO, achieving a selectivity of 92.6% for CO under full-spectrum Xe-lamp irradiation (Figure 13b,c), the surface temperature of the Co@NC-700 catalyst rose rapidly under the irradiation of the full spectrum, and finally stabilized at 400 °C. Owing to the coating effect of the carbon shell, the Co@NC-700 catalyst showed excellent stability over 26 consecutive cycles, outperforming Co NPs are loaded onto graphitic carbon and bare Co NPs (Figure 13e). Moreover, carbon encapsulation significantly lowers CO adsorption energy on Co_9_@graphene, and thus enhances CO desorption during photocatalytic CO_2_ hydrogenation (Figure 13f). This study presents a straightforward and effective approach for harnessing abundant and clean solar energy to efficiently facilitate CO_2_ conversion.

### 3.4. Photocatalytic Contaminant Degradation

Pollutants, which encompass dyes, antibiotics, and heavy metals in aquatic environments, as well as volatile organic compounds (VOCs) and other hazardous gases in the atmosphere, not only jeopardize the natural ecosystem but also threaten the health of both animals and humans; therefore, it is imperative to urgently remove these pollutant molecules from our environment. Among the many pollution control technologies, photocatalysis has emerged as a prominent area of research within the domain of pollution mitigation, due to its advantages of green, economic, and depth treatment. Up to now, MOFs have been considered as good candidates for the photocatalytic degradation of pollutants due to their potential advantages, which have been mentioned above. Based on this, MOFs are widely utilized for the degradation of organic and other toxic pollutants [76,77,78,79,80].

**Figure 14:**
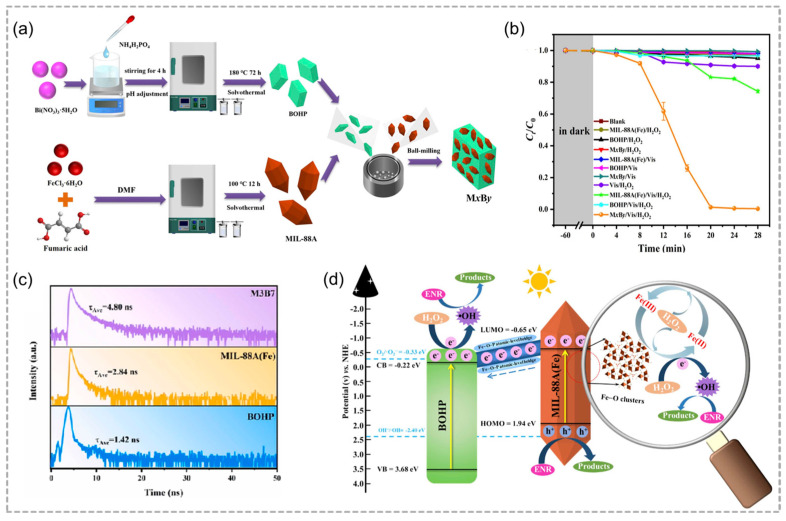
(**a**) Schematic presentation of the preparation route of MxBy heterojunctions. (**b**) Temporal analysis of photo–Fenton degradation of ENR across various reaction systems. (**c**) Time–resolved PL decay spectra. (**d**) Possible ENR catalytic degradation mechanism. Adopted with permission from Elsevier [56].

Recently, Wang and co-workers prepared MIL-88A(Fe)/BOHP (MxBy) heterojunctions by the ball-milling method (Figure 14a) [56]. The interaction between MIL-88A(Fe) and BOHP not only significantly reduces the transfer energy barrier of photogenerated carriers, but also shortens the migration distance (Figure 14c). Consequently, the most suitable reaction system (M3B7/Vis/H_2_O_2_) exhibited outstanding performance in the photo-Fenton degradation of ENR, achieving nearly 100% catalytic efficiency and exhibiting significant mineralization capacity within a duration of 28 min under visible light (Figure 14b,d). In addition, it can be seen from the results of toxicity evaluation and economic analysis that the M3B7/Vis/H_2_O_2_ system is environmentally friendly and energy-saving.

**Figure 15:**
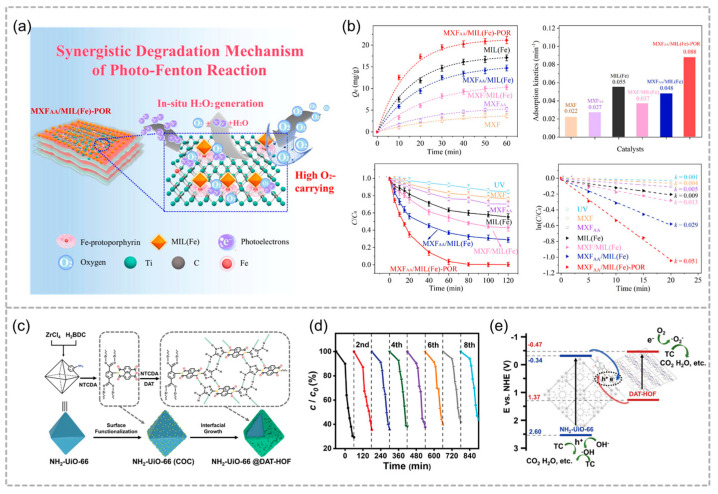
(**a**) Schematic diagram of TCL degradation by MXFAA/MIL(Fe)-POR photo–Fenton. (**b**) Photo–Fenton TCL degradation performance. Adopted with permission from Elsevier [81]. (**c**) Schematic diagram of the synthesis route of NH_2_–UiO–66@DAT–HOF photocatalysts. (**d**) Efficiency of TC photodegradation in the presence of the U@H2 hybrid. (**e**) Energy band structure of the U@H2 heterojunction. Reproduced with permission from WILEY [57].

Thiacloprid (TCL) is a representative class of neonicotinoid insecticide, which is not only relatively stable and hard to decompose but also has high solubility and toxicity, which will result in considerable environmental degradation and present serious threats to human health. Zhao and co-workers developed a Ti_3_C_2_ MXene/MIL-100(Fe) hybrid to in situ generate H_2_O_2_ for the photo-Fenton degradation of thiacloprid (Figure 15a) [81]. A heterojunction has been established between MXene and MIL-100(Fe), and combined with Fe-protoporphyrin’s biomimetic oxygen transport, it creates a synergistic system that significantly enhances H_2_O_2_ production through efficient charge carrier separation. As expected, MXene/MIL-100(Fe) efficiently removed TCL with 80% TOC in 120 min and demonstrated over 97% degradation stability across ten successive uses, outperforming many reported photo-Fenton catalysts by a factor of 21 to 60 in H_2_O_2_-free systems (Figure 15b).

Tetracycline antibiotics belong to a category of broad-spectrum antibiotics, which are biologically toxic to aquatic organisms and terrestrial animals through the food chain. Ultimately, the potential risks to human health are enormous. Therefore, it is crucial to eliminate tetracycline antibiotic residues from the environment to protect both ecological integrity and human health. Recently, Ma and co-workers employed stable HOFs to fabricate a core–shell composite (Figure 15c) [57], the NH_2_-UiO-66@DAT-HOF with distinct nanostructure shows outstanding stability and excellent photocatalytic efficiency for tetracycline degradation, outperforming its parent materials by 60.7 and 7.6 times (Figure 15d). The improved photocatalytic efficiency is mainly attributed to the unique structure of the DAT-HOF shell surrounding the MOFs’ core, which broadens visible light utilization and improves hole–electron pair separation through an S-scheme heterojunction (Figure 15e).

Regarding the cost and stability of catalysts, various methods such as developing eco-friendly synthesis approaches, using earth-abundant metals and low-cost linkers, incorporated with cheap support materials and designed with recyclable MOFs can reduce the cost of MOF production and enhance photocatalytic efficiency. Furthermore, various approaches like using robust metal clusters and stable linkers, functionalizing linkers, integrating with stable materials, and coating or encapsulating MOFs can enhance their stability in different conditions. This paves the way for cost-effective and robust MOF-based photocatalysts tailored for sustainable pollutant removal.

### 3.5. Photocatalytic Fixation of Nitrogen to Ammonia

Ammonia (NH_3_) is a basic industrial chemical related to the national economy and people’s livelihoods, and is widely used in fertilizer, environmental protection, military, and other fields. Furthermore, NH_3_ synthesis technology serves a critical function as an intermediary for an efficient hydrogen storage medium and carbon neutral carrier [82]. However, the traditional Haber–Bosch process cannot meet the growing need for cleaner, greener, and sustainable alternatives, due to its high operating conditions (high temperature: 350–550 °C and high pressure: 150–350 atm) [83,84,85]. A photocatalytic nitrogen reduction reaction (NRR) holds remarkable potential by harnessing solar light to enhance carrier separation efficiency within the catalyst, thereby facilitating the transformation of N_2_ molecules into NH_3_ or N_2_H_4_ under ambient conditions. All in all, MOFs have the potential to improve NRR efficiency by combining high N_2_ adsorption capacity, rapid kinetics of N_2_ activation, and excellent photoactivity.

**Figure 16:**
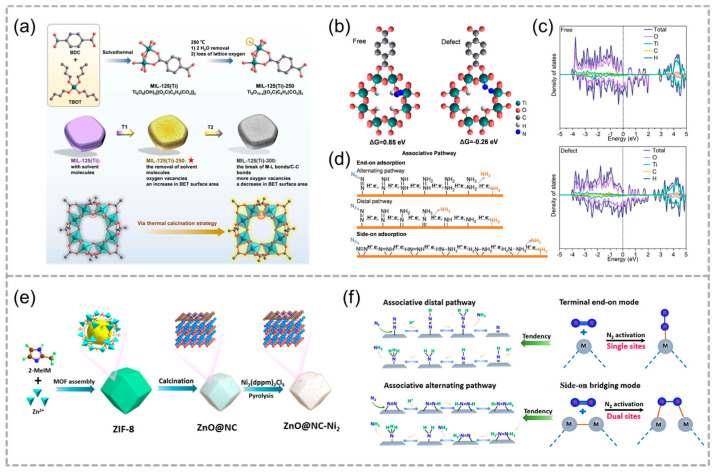
(**a**) The synthesis of MIL–125(Ti) involves a solvothermal method followed by thermal calcination. T1 and T2 denote the different calcination temperature. MIL–125(Ti)–250 with oxygen vacancies and an increase in BET surface area. (**b**) The adsorption of an N_2_ molecule on both free and defect modes, along with the associated binding energy. (**c**) Density of states of free and defect structure. (**d**) The associative pathway encompasses both “end–on” (vertical) and “side–on” (parallel) configurations for N_2_ adsorption. Reproduced with permission from WILEY [86]. (**e**) Schematic illustration for the construction of ZnO@NC-Ni2 with dinuclear Ni2 sites based on ZIF–8. (**f**) Schematic diagram of possible pathways for N_2_ reduction to NH_3_. Adapted with permission from ACS [62].

An improved N_2_ adsorption capacity and thus enhanced photocatalytic NH_3_ yield is the main purpose. Oxygen vacancy could facilitate the adsorption and activation of N_2_ molecules. Based on this idea, Pang and co-workers adopted a low-temperature thermal calcination strategy to modulate the electronic structure of MIL-125(Ti)-250 and increase surface-active sites (Figure 16a) [86]. The abundant oxygen vacancies and high specific surface area synergistically facilitate the N_2_ molecules’ adsorption and activation, and MIL-125(Ti)-250 with a higher concentration of exposed active sites exhibited a twice-higher NH_3_ formation rate than that of the parent MIL-125(Ti), a higher Ti^3+^/Ti^4+^ atomic ratio improves the carrier separation and boosts the efficiency of photocatalytic NH_3_ synthesis (Figure 16b–d).

Directly introducing highly active sites to enhance the efficiency of photocatalytic NRR is desired. Single-atom catalysts (SACs) exist in the form of single-atom dispersion. By maximizing the utilization of active metal sites, SACs typically demonstrate exceptional catalytic activity. Han and co-workers engineered a dinuclear Ni2 site-modified ZnO@NC heterojunction for efficient N_2_ photofixation under mild conditions (Figure 16e) [62], which possessed a NH_3_ yield of 70.3 μg gcat^−1^ h^−1^, significantly surpassing ZnO@NC and ZnO@NC-Ni1 yields. The binuclear Ni2 active sites in ZnO@NC-Ni2 facilitate photocatalytic N_2_ reduction by lowering the activation energy through an associative alternating pathway (Figure 16f). Recently, Meng and co-workers created electron–metal-support interactions (EMSI) by depositing Ru single atoms onto UiO-66 through covalent bonding [87]. The photocatalytic ammonia production rate increased significantly from 4.57 to 53.28 μmol g^−1^ h^−1^ by introducing defects in UiO-66 and loading Ru single atoms, with the Ru atom enhancing N_2_ reduction due to its electron-rich state and strong reducing ability.

## 4. Summary and Outlook

The distinctive characteristics of MOF-based materials, including their diverse structures, tunable pore size, high porosity, and large surface areas, undoubtedly endow them with significant potential in catalysis and have garnered them considerable attention in recent years. In this review, we discuss various MOF composites, and recent applications in solar-driven CO_2_ conversion, water splitting (HER and OER), contaminant degradation, and nitrogen fixation to ammonia. Despite the unique structure and physicochemical properties of MOFs, they have shown great potential in the field of photocatalysis, several challenges including limited active sites, low solar-energy-utilization efficiency, poor cycle stability and product selectivity, hindering their widespread use in photocatalysis. Therefore, for the future advancement of MOFs in photocatalytic applications, it is essential to tackle the following challenges:

(1) The majority of photocatalysts can effectively absorb ultraviolet (UV) or visible (Vis) light, whereas the near-infrared (NIR) spectrum, which constitutes approximately 50% of natural sunlight, has seldom been harnessed. Introducing the long-wavelength-light-responsive unit into MOFs and thus extending the region of harvesting light to visible or NIR light is appealing.
(2) Improving two advantageous redox half reactions by concurrently utilizing electrons and holes presents considerable promise from a practical viewpoint. The efficiency of photocatalytic water decomposition of MOF materials to produce hydrogen, oxygen, and total water is still low. Although the photocatalytic water decomposition activity of some MOFs has been improved by a series of means, it is still lower than that of traditional inorganic semiconductors and significant advancements are required before practical implementation can be achieved. The follow-up work still needs to develop specific methods to enhance the photocatalytic activity of MOFs. Creating the heterojunctions’ internal electric fields at material interfaces not only promotes efficient charge carrier separation and transfer but also broadens the absorption spectrum and thus boosts photocatalytic activity. MOF-based heterojunctions constitute a versatile platform for promoting next-generation photocatalytic and optoelectronic technologies, offering solutions to energy and environmental issues.
(3) At present, the photoreduction reaction of CO_2_ in the laboratory is mainly completed under the high-purity CO_2_ atmosphere, while the CO_2_ content in the actual industrial exhaust gas is only 5–15%, even the CO_2_ concentration in air is only 400 ppm. In this case, minimizing the high energy consumption associated with the CO_2_ purification process holds considerable scientific importance and directly realizes the photoreduction reaction of low-concentration CO_2_ in practical application research. The development of high-efficiency catalysts that can effectively convert low-concentration CO_2_ into desired products is a key step to realize the resource utilization of CO_2_ in the future.
(4) Through the artificial photosynthesis process directly driven by natural light, coupling the photocatalytic CO_2_ reduction and H_2_O oxidation to construct a two-in-one photocatalytic system directly captures CO_2_ and reduces it into fuel or high value-added chemicals in situ, accompanied by the release of oxygen. This approach presents both challenges and opportunities for achieving carbon resource recycling, as it not only significantly reduces the concentration of atmospheric CO_2_ but also generates high value-added chemicals.
(5) In terms of contaminant degradation, numerous studies have focused on photocatalytic oxidation to eliminate highly concentrated and resistant organic compounds (like dyes) from industrial wastewater; however, there has been limited research on the treatment of ultralow concentration organic pollutants in source water.

Although the progress on MOF photocatalysis is still in its nascent stage, the efforts of researchers in tackling the several challenges continue. In the future, we firmly believe that MOF-based materials will undoubtedly attract more attention and continue to unveil captivating surprises in catalysis.
